# The protective effect of Malva sylvestris on rat kidney damaged by vanadium

**DOI:** 10.1186/1476-511X-10-65

**Published:** 2011-04-23

**Authors:** Wafa Marouane, Ahlem Soussi, Jean-Claude Murat, Sofiane Bezzine, Abdelfattah El Feki

**Affiliations:** 1 Laboratoire d'Ecophysiologie Animale, Faculté des Sciences, Route de Soukra 3038 Sfax - University of Sfax-Tunisia; 2 Laboratoire de Biochimie et de Génie Enzymatique des Lipases, Ecole Nationale d'Ingénieurs, Route de Soukra 3038 Sfax - University of Sfax-Tunisia; 3 Laboratoire de Biochimie, Institut Supérieur de Biotechnologie de Sfax, Route de Soukra 3038 Sfax - University of Sfax-Tunisia; 4 Laboratoire de Biologie Cellulaire, Faculté de Médecine, Toulouse, France

**Keywords:** Ammonium metavanadate, Malva sylvestris, nephrotoxicity, oxidative stress, antioxidant enzymes

## Abstract

**Background:**

The protective effect of the common mallow (Malva sylvestris) decoction on renal damages in rats induced by ammonium metavanadate poisoning was evaluated. On the one hand, vanadium toxicity is associated to the production of reactive oxygen species, causing a lipid peroxidation and an alteration in the enzymatic antioxidant defence. On the other hand, many medicinal plants are known to possess antioxidant and radical scavenging properties, thanks to the presence of flavonoids. These properties were confirmed in Malva sylvestris by two separate methods; namely, the Diphenyl-2-picrylhydrazyl assay and the Nitroblue Tetrazolium reduction assay.

**Results:**

In 80 rats exposed to ammonium metavanadate (0.24 mmol/kg body weight in drinking water) for 90 days, lipid peroxidation levels and superoxide dismutase, catalase and glutathione peroxidase activities were measured in kidney. A significant increase in the formation of free radicals and antioxidant enzyme activities was noticed. In addition, a histological examination of kidney revealed a structural deterioration of the renal cortical capsules and a shrinking of the Bowman space. In animals intoxicated by metavanadate but also given a Malva sylvestris decoction (0.2 g dry mallow/kg body weight), no such pathologic features were observed: lipid peroxidation levels, antioxidant enzyme activities and histological features appeared normal as compared to control rats.

**Conclusion:**

Malva sylvestris is proved to have a high antioxidative potential thanks to its richness in phenolic compounds.

## Background

Vanadium is a naturally occurring ubiquitous transition metal usually found in high concentrations in the earth's crust, oceans, soil and fossil fuels [1]. It is also widely used primarily in the manufacture of corrosion-resistant alloys [1,2]. Environmental pollution due to vanadium is a potential health threat since soluble vanadium salts from μM to mM range were found to exert adverse effects mainly on liver and kidney [3]. Vanadate ions were also shown to possess regulatory properties in the cell metabolism. Ammonium metavanadate was found to mimic all or most of the actions of insulin in intact cell systems by inhibiting a tyrosine-specific phosphoprotein phosphatase [4]. Besides affecting numerous biological mechanisms, Vanadate oligomers also interact with several proteins such as membrane-bound transport systems [5,6].

As is the case with many other heavy metals, vanadium poisoning results in the production of reactive oxygen species (ROS), causing a peroxidation of structural lipids and an alteration of the antioxidative activity of enzymes, namely, superoxide dismutase (SOD), catalase (CAT) and glutathione peroxidase (GPX) [7].

Several studies confer to flavonoids and other plant-derived polyphenolic compounds antioxidant and free radical scavenging properties [8]. Flavonoids are a group of naturally occurring compounds widely distributed as secondary metabolites in plants [9]. These natural compounds exert a variety of biological and chemical activities. As antioxidants, the flavonoids inhibit the lipid peroxidation induced by various prooxidants in liver homogenates, microsomes, mitochondria and liposomes. These properties are related to the ability of flavonoids to chelate metal ions and to scavenge singlet oxygen, superoxide anions, peroxyl radicals, hydroxyl radicals and peroxynitrite [10,11]. Flavonoids are therefore considered to be the active ingredients in several medicinal plants [12].

Malva sylvestris, a traditional medicinal plant, was used in traditional phytotherapy and cosmetic treatments [13]. Fluid extract of Malva sylvestris flowers and leaves are used as a valuable remedy for cough and inflammatory diseases of mucous membranes [14]. The biological activity of this plant may be attributed to antioxidants, such as polyphenols, vitamin C, vitamin E, β-carotene, and other important pythochemicals [15]. In a previous investigation, gossypetin 3-sulphate-8-O-β-D-glucoside and hypolaetin 3'-sulphate were identified as the major flavonoid constituents in the leaf tissue of Malva sylvestris [16].

Other compounds with chemotaxonomic significance for the Malvaceae are the 8-hydroxyflavonoids so far the isolation of three 8-hydroxyflavonoid sulphates has been reported from Malva sylvestris leaves [17].

A comparative study of the composition in nutraceuticals (phenolics, flavonoids, carotenoids, ascorbic acid, tocopherols, sugars, and fatty acids) and antioxidant properties of different parts of Malva sylvestris (leaves, flowers, immature fruits, and leafy flowered stems) was evaluated by Barros et al [15] in order to valorise all the plant as functional food or even pharmafood.

The renal tissue is known to accumulate heavy metals including vanadium, which are reported to be responsible for chronic renal diseases [18].

The aim of this study is therefore to determine the antioxidant capacity of Malva sylvestris and to evaluate the protective effect of this plant against vanadium-induced lipid peroxidation and antioxidant enzyme alterations in the kidney of rats fed on 15% proteins commercial food pellets.

## Results

### Antioxidant and free radical scavenging activities of Malva sylvestris decoction

To evaluate the potential antioxidant activity of Malva sylvestris decoction, we used the Nitroblue Tetrazolium reduction assay. Percentage of antioxidant activity of the decoction was calculated using the formula: Antioxidant activity (%) = ([A_0 _- A_1_]/A_0_] × 100.

A_0 _is absorbance of blank at 560 nm,

A_1 _is absorbance of the sample in the presence of the decoction.

Malva sylvestris decoction inhibited the development of the colour associated with the suppression of the ROS generated in the photochemical system. Calculated IC_50 _value is 1 g of dry Malva sylvestris/l (Table 1).

**Table 1:** Rate of inhibition of superoxide anion radicals by Malva sylvestris decoction

Dry Malva sylvestris decoction concentration (g/l)	0,006	0,01	0,02	1
Inhibition %	19,15	23,75	26,87	49,37

The antiradical activities of the decoction were determined using the DPPH free radical assay.

Inhibition of free radical DPPH, in percentage was calculated as:

Scavenging activity (%) = ([A_0 _- A_1_]/A_0_] × 100.

A_0 _is absorbance of blank at 517 nm,

A_1 _is the absorbance of the sample in the presence of the decoction.

IC50 in this test was defined as the concentration of Malva sylvestris decoction that was able to inhibit 50% of the total DPPH radicals.

As shown in table 2, the preparation was able to reduce the stable free radical DPPH to the yellow- color with an IC_50 _= 0.68 g of dry Malva sylvestris/l.

**Table 2:** Free radical scavenger effect of Malva sylvestris decoction

Dry Malva sylvestris decoction concentration (g/l)	0,005	0,01	0,68	1
Inhibition %	13,75	22,5	50	72,5

### Protective effects of Malva sylvestris in vivo

#### Lipid peroxidation

**Figure 1:**
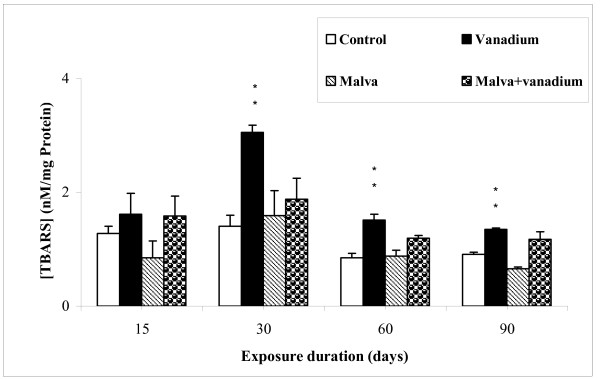
**Lipid peroxidation levels (TBARS) in kidney of controls and treated rats during 15, 30, 60 and 90 days**. Values are the mean ± SEM, normalized to 100 for controls (n = 5). Student's t-test was used for comparison: * (p < 0.05), ** (p < 0.01).

Lipid peroxidation levels in kidney of rats subjected to the different treatments was measured according to Yagi method [19]. TBARS level are shown in Figure 1.

As compared to controls, lipid peroxidation was significantly increased at day 30 (by about 117.5%) in vanadium-treated animals. This increase indicates an enhancement of membrane lipid peroxidation after 30 days of treatment. However, no change was observed in vanadium-treated rats given the Malva sylvestris decoction.

#### Kidney histopathology

**Figure 2:**
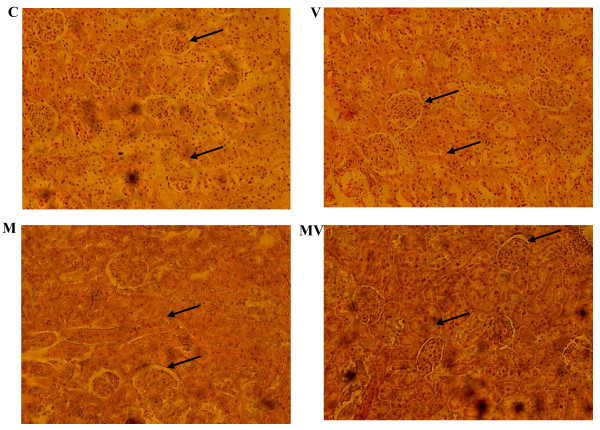
**Micrographs of renal sections from controls and treated rats during 30 days**. C: control (normal) rats; V: rats treated with vanadium; M: normal rats given Malva sylvestris decoction; MV: vanadium treated rats given Malva sylvestris decoction. (n = 5). Arrows indicate Bowman's space and oedema of interstitial cells.

The morphologic changes induced in the kidney of treated rats were evaluated after hematoxyline-eosine coloration. Results are presented in Figure 2. Under our experimental conditions, metavanadate poisoning induces 1) a structural deterioration of the renal cortical capsules, 2) a decrease of the Bowman's space, and 3) a hypertrophy of interstitial cells of the limiting membrane of proximal tubules resulting in a reduction of the urinary space. Malva sylvestris decoction appears to prevent these histopathological features.

#### Serum creatinine level

**Figure 3:**
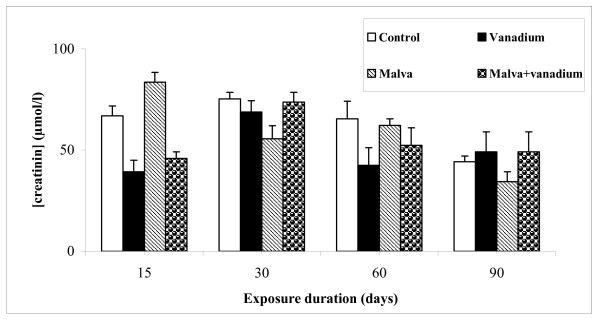
**Creatinine level in serum of control and treated rats during 15, 30, 60 and 90 days**. Values are the mean ± SEM, normalized to 100 for controls (n = 5). Student's test was used for comparison

Serum creatinine concentration was detected using commercial kits bought from *Biomagreb *(Ref: 20151). The creatinine level in plasma of different treated rats is shown in Figure 3.

Our results showed no significant difference concerning the serum creatinine level compared to the different experimental groups.

#### Antioxidant enzyme activities

**Figure 4:**
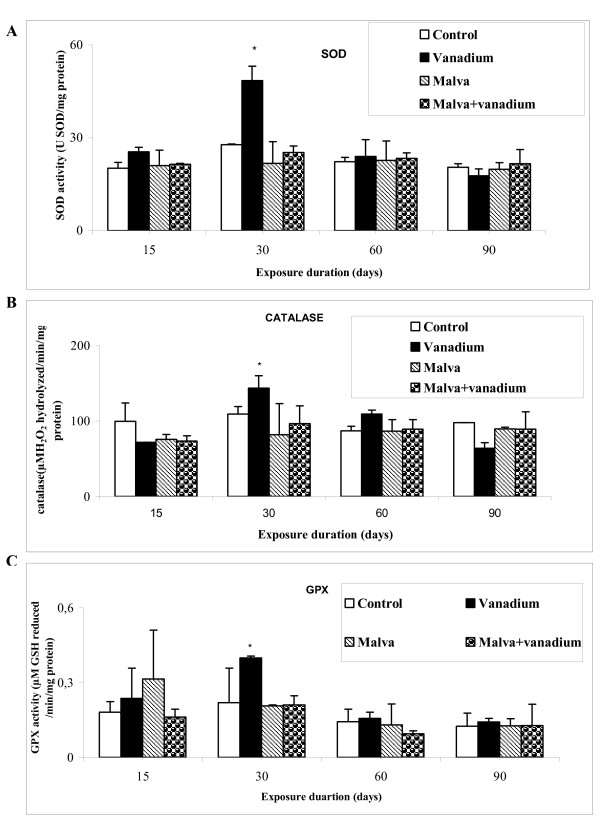
**Activities of some enzymes in controls and treated rats after 15, 30, 60 and 90 days**. (A) Superoxide dismutase (SOD), (B) catalase (CAT) and (C) Glutathione peroxidase (GPX). Values are the mean ± SD, normalized to 100 for controls (n = 5). Student's test was used for comparison: * (p < 0.05)

Activities of some major enzymes involved in the defence against oxidative stress, SOD, CAT and GPX, were measured in control and treated rats using the methods of Asada, Aebi and Paglia respectively [20-22]. As shown in Figure 4, vanadium poisoning resulted in an increased activity of kidney SOD, CAT and GPX activities at day 30 of treatment with 74.5%, 31.5% and 82% respectively.

However, SOD, CAT and GPX activities in rats drinking the Malva sylvestris decoction were similar to control values vanadium-poisoned or not.

## Discussion

The aim of this study was to evaluate the antioxidant potency of Malva sylvestris decoction in vitro and to examine its protective effect against vanadium-induced lipid peroxidation and antioxidant enzyme alterations in rat kidney.

Among the various methods used to evaluate the total antioxidant activity of natural compounds, the DPPH radical scavenging and the NBT reduction assays are the most commonly used. Both methods were used in the present study. Malva sylvestris decoction shows a notable DPPH radical scavenging activity (IC50 = 0.68 g of dry Malva sylvestris/l) which is attributed to its hydrogen donating ability. DPPH has been widely used to evaluate the free radical-scavenging effectives of various antioxidant substances [23]. Italian authors reported, in a study with aerial parts of Malva sylvestris, 24% of DPPH scavenging activity at 20 mg/l [24]. Other report on mallows from Turkey revealed scavenging effects on hydrogen peroxide of 46.19% at 5 g/l [25]. Annan and Houghton indicated that quantitative DPPH test on Malvaceae species: Gossypium arboretum revealed antioxidant properties, with IC50 of 35.7 mg/l [26].

The NBT reduction assay is also positive. Malva sylvestris decoction was able to inhibit superoxide anion radical with an IC_50 _= 1 g of dry Malva sylvestris/l. These findings suggest that flavonoids from Malva sylvestris are responsible for the antioxidant effect. Flavonoids widely distributed in plants have the ability to scavenge superoxide and hydroxyl radicals by single-electron transfer [27]. It is also worth noting that antioxidant defence systems have coevolved with the aerobic metabolism to counteract oxidative damages from ROS [28].

Toxicity of many metals, including vanadium, is associated with the increased production of ROS leading to an oxidative stress in cells [29]. An excessive level of lipid peroxidation, expressed by thiobarbituric acid reactive substances (TBARS) accumulation, is now recognized to be a critically important phenomenon resulting from the oxidative stress [30].

Under our experimental conditions, TBARS level increase in kidney of vanadium-treated rats at day 30, as compared to control rats. Other authors reported that an increase of spontaneous TBARS formed in kidney of rats after vanadium administration was also observed [31]. Previous studies reported that vanadium ingested by rats in drinking water for 4 weeks significantly stimulated TBARS production in kidney in the presence of exogenous Fe^2+ ^[32]. Oxidative stress induced by an acute exposure (1 and 10 days) to an ammonium metavanadate concentration of 5 mg*/*kg body weight was studied by Soussi A et al [33]. TBARS were significantly increased by 197% compared to control.

Oral administration of Malva sylvestris decoction prevents this vanadium-induced increase of TBARS level. Pharmaceutical development of flavonoids usually involves several potential activities. Phenoxy radicals are readily formed by donation of phenolic hydrogens and subsequently react with peroxy radicals involved in lipid peroxidation. Flavonoids and their metabolites have one-electron reduction potentials lower than those of highly oxidising reactive oxygen species (ROS), thus they are capable to reduce these species [34].

The biological effects of ROS are controlled in vivo by a wide spectrum of enzymatic and non-enzymatic defence mechanisms such as SOD which catalyzes the dismutation of superoxide anions into hydrogen peroxide, CAT which detoxifies H_2_O_2 _and GPX which converts hydroperoxides into nontoxic alcohols [35].

Vanadium poisoning is reported to alter the activity of several enzymes involved in defence against an oxidative stress [36]. In the present study, vanadium was found to enhance SOD, GPX and CAT activities on day 30. These effects are reversed by the treatment with the Malva sylvestris decoction. Available literature presents abundant data about the effect of inorganic and organic vanadium compounds administered to rats on the activity of some antioxidant enzymes in liver and/or kidney [37,38]. SOD and CAT are proved to be the major enzymes playing a role in the elimination of ROS derived from redox processes [28]. The studies of Sciobor et al [39] have demonstrated lack of a direct effect of vanadate and vanadyl on the activity of the main antioxidant enzymes (SOD and CAT), suggesting that many biological and toxicological effects of vanadium may be mediated by oxidative reactions of the metal or of its complexes formed with physiologically relevant biomolecules, rather than by direct modulation of enzymatic activities. It was elsewhere reported that the increase of SOD activity counteracts some adverse effects of vanadium [40]. Again, in animals given the Malva sylvestris decoction, the vanadium-induced increase of SOD activity is avoided. Previous studies have shown that Malvaceae species: Hibiscus sabdariffa have antioxidant properties which may probably be due to its free radical scavenging ability [41]. Other authors reported that CAT and SOD activities were restored to normal after treatment with Malvaceae species: Thespesia populnea [42]. Flavonoids, like other phenolic compounds with free hydroxyls, are considered to possess some degree of antioxidant activity. In fact, a range of flavonoid-enriched plant extracts is widely marketed for their antioxidant properties and putative health benefits [34].

Histological examination of kidney shows that vanadium induces a structural deterioration of the renal cortical capsules followed by a decrease of the Bowman's space. Moreover, this metal produces a hypertrophy of interstitial cells of the limiting membrane in proximal tubules, therefore reducing the urinary space. There is however no statistically significant change in the serum creatinine level, as compared to controls; which indicates that the nephropathy was not yet severe. These results are in agreement with those obtained by De la Torre et al [18] who reported that Vanadium, as many other metals, tends to accumulate in the kidney predisposing to nephrotoxicity. Data presented by Al-Bayati et al [43] revealed that vanadate treatment affected both cortical and medullary regions of the kidney and produced moderate to severe damage leading to fibrosis of both regions. Multifocal lesions were observed in the cortex and medulla at 12 and 25 days after the last injection of vanadate, with the cortex being more affected than the medulla. Mohamed et al [44] reported that kidney is vulnerable to damage because of larger perfusion and the increased concentration of excreted compounds that occurs in renal tubular cells. However, the Malva sylvestris decoction reduces the abnormal features observed in kidney slices from vanadium-poisoned rats. The nephroprotective mechanism appears to be through modulation of various anti-oxidant parameters thereby improving the overall anti-oxidant defence of the renal tissue [45]. It was reported previously that kidney histopathological were decreased in Hibiscus sabdariffa treated rats [46].

Most of the beneficial effects of the administration of the Malva sylvestris decoction are due to the presence of flavonoids, which are phenolic compounds. Flavonoids interfere with the oxidation process by 1) reacting with free radicals, 2) chelating catalytic metals and 3) acting as oxygen scavengers [47-49].

From our work, it can be concluded that the Malva sylvestris decoction, rich in phenolic compounds, possesses a potent antioxidant activity, enough to prevent vanadium-induced nephrotoxicity in our experimental model. It seems very important to suggest that Malva sylvestris could therefore be considered as a valuable therapy used in diabetic and inflammated rats. This constitutes the aim of unpublished work.

## Materials and methods

### Preparation and characterization of the Malva sylvestris decoction

Fresh Malva sylvestris leaves and flowers were dried and then powder-grinded. 1 g of the obtained dry powder was boiled for 10 min in1 l of water and then filtered.

The free radical scavenger activity of the Malva sylvestris decoction was determined by the 1,1-Diphenyl-2-picrylhydrazyl (DPPH) assay, as specified by Koleva *et al. *[50]. 1 ml of 60 μM DPPH in ethanol was added to 1 ml of different concentrations of the decoction. After 30 min of incubation at room temperature, the absorbance was read at 517 nm.

The inhibition of Nitroblue Tetrazolium (NBT) reduction by photochemically generated O^-^_2 _[51] was used to determine the superoxide anion scavenging activity. The reaction mixture contained 100 μl of Malva sylvestris decoction added to 800 μl of phosphate buffer (pH 7.4) containing 100 μl of 96 μM NBT, 100 μl of 6.5 mM Ethylenediaminetetraacetic acid (EDTA) and 50 μl of 4 μM riboflavin. After 10 min exposure to light, the absorbance was read at 560 nm. IC_50 _value was defined as the concentration (g of dry Malva sylvestris leaves and flowers/l) which decreases absorbance by 50%.

### In vivo studies

#### Animals and experimental procedure

Two-month old and about 160 g body weight Wistar male rats supplied by Central pharmacy from Tunisia (80 rats) and fed on 15% proteins food pellets (*SICO, Sfax, Tunisia*). 20 g/animal/day were used in the present study. The animals were housed in cages and maintained in a controlled environment (23°C and stable humidity) in an animal house with a constant 12 h light and 10 h darkness cycle. Control animals (C) were kept on tap water. A group (V) was given a solution of ammonium metavanadate as sole beverage (1.185 mmol/l corresponding to 60 mg ammonium metavanadate/kg body weight), another group (M) was given the Malva sylvestris decoction resulting in an intake of 0.2 g of dry plant/kg body weight/day, and a group (MV) was given the Malva sylvestris decoction (similarly to group M) containing 1.185 mmol/l of ammonium metavanadate.

All treatments were monitored during 15, 30, 60 and 90 days. On day 90, animals were sacrificed and kidney was fixed into Bouin's fixative, embedded into paraffin, cut into 5-μm sections and stained with hematoxyline-eosine for light microscopy.

#### Chemical analyses

Serum creatinine concentration was determined by using ready-made commercial kits bought from *Biomagreb *(Ref: 20151). Creatinine reacts with picric acid in an alkaline medium to develop a complex which absorbs at 492 nm.

Proteins were estimated by the Lowry's method [52] using bovine serum albumin as a standard.

#### Estimation of lipid peroxidation levels in kidney

The lipid peroxidation level in kidney was determined by the method of Yagi [19]. Briefly, a kidney sample of about 1 g was cut into small pieces and homogenized in 2 ml of ice-cold Tris Buffered Saline (TBS) with pH 7.4, centrifuged at 9000 × g (4°C, 20 min). Supernatants were collected and stored at -80°C until use.

125 μl of supernatants were mixed with 50 μl of TBS and 125 μl of 20% Trichloroacetic Acid (TCA) containing 1% Butylated hydroxytoluene (BHT) to precipitate proteins and centrifuged at 1000 × g (4°C, 10 min). 200 μl of the new supernatants were then mixed with 40 μl of HCl (0.6 M) and 160 μl of 120 mM Thiobarbituric Acid (TBA) dissolved in 26 mM trishydroxymethyl aminomethane (Tris) and the mixture was heated at 80°C during 10 min. The absorbance of the resulting supernatants was measured at 530 nm.

#### Antioxidant enzyme assay

##### Superoxide dismutase activity

SOD activity was evaluated by the photoreduction of NBT [20]. In this assay, one unit of SOD is defined as the amount inhibiting the photoreduction of NBT by 50%.

##### Catalase activity

CAT activity was measured by the method described by Aebi [21]. The used reaction mixture (1 ml) contained 100 mM of phosphate buffer (pH 7.4), 50 mM of H_2_O_2 _and kidney homogenate.

The reaction started by adding H_2_O_2 _and its decomposition was observed by following the decrease in absorbance at 240 nm for 1 min.

##### Glutathione- Peroxidase activity

The GPX activity was determined in the kidney cytosolic fraction according to the method of Paglia and Valentine [22]. Tissue homogenate was mixed with 400 μl of 0.1 mM glutathione (GSH) and 200 μl of 67 mM of KNaHPO4 (pH = 7.8). After 5 min of preincubation at 25°C, 200 μl of 1.3 M of H_2_O_2 _was added. After 10 min, the mixture was treated with 1 ml of 1% TCA and centrifuged at 3000 × g and 4°C during 10 min.

Supernatants were homogenized with 0.32 M of Na_2_HPO_4 _and 1 mM of 5,5'-Dithio-bis(2-nitrobenzoic acid) (DTNB). The enzyme activity was spectrophotometrically measured at 412 nm and expressed as μmoles of reduced-GSH/mn/mg protein.

### Statistical analysis

Student's t-test was used for the comparison of means (n = 5). p < 0.05 was considered to demonstrate a significant difference between values.

## Abbreviations

ROS: reactive oxygen species; SOD: superoxide dismutase; CAT: catalase; GPX: glutathione peroxidase; DPPH: 1-Diphenyl-2-picrylhydrazyl; NBT: Nitroblue Tetrazolium; EDTA: Ethylenediaminetetraacetic acid; TBS: Tris Buffered Saline; TCA: Trichloroacetic Acid; BHT: Butylated hydroxytoluene; TBA: Thiobarbituric Acid; TRIS: trishydroxymethyl aminomethane; GSH: glutathione; DTNB: 5,5'-Dithio-bis(2-nitrobenzoic acid); TBARS: thiobarbituric acid reactive substances.

## Competing interests

The authors declare that they have no competing interests.

## Authors' contributions

WM and AS carried out all the studies, analyzed the data and drafted the manuscript. JCM helped with the analysis of the data and to correct the manuscript. SB participated in the study design and helped to draft the manuscript. AF helped with the discussion of the data. All authors have read and approved the final manuscript.
